# The genome sequence of the Autumn Spider, *Metellina segmentata* (Clerck, 1757)

**DOI:** 10.12688/wellcomeopenres.19435.1

**Published:** 2023-05-16

**Authors:** Sergio Henriques, Olga Sivell, Natural History Museum Genome Acquisition Lab, Darwin Tree of Life Barcoding collective, Wellcome Sanger Institute Tree of Life programme, Wellcome Sanger Institute Scientific Operations: DNA Pipelines collective, Tree of Life Core Informatics collective, Darwin Tree of Life Consortium

**Affiliations:** 1 Global Center for Species Survival, Indianapolis Zoo, Indianapolis, Indiana, USA; 2 Natural History Museum, London, England, UK

**Keywords:** Metellina segmentata, Autumn spider, genome sequence, chromosomal, Aranae

## Abstract

We present a genome assembly from an individual male *Metellina segmentata* (the Autumn spider; Arthropoda; Arachnida; Aranae; Tetragnathidae). The genome sequence is 1,665.1 megabases in span. Most of the assembly is scaffolded into 13 chromosomal pseudomolecules, including the X1 and X2 sex chromosomes. The mitochondrial genome has also been assembled and is 17.8 kilobases in length.

## Species taxonomy

Eukaryota; Metazoa; Ecdysozoa; Arthropoda; Chelicerata; Arachnida; Araneae; Araneomorphae; Entelegynae; Araneoidea; Tetragnathidae; *Metellina*; *Metellina segmentata* (Clerck, 1757) (NCBI:txid94026).

## Background

*Metellina segmentata* (Clerck, 1757), commonly known as the Autumn spider, is a species from the family Tetragnathidae, long-jawed orb weavers. It is one of three *Metellina* species occurring in Britain, with a characteristic “tuning fork” pattern on the carapace (around fovea). *Metellina segmentata* is similar in appearance to the two other British members of the genus, *M. merianae* (Scopoli, 1763) and *M. mengei* (Blackwall, 1869) ( Roberts, 1995). All three species are variable in colouration and markings. *M. merianae* can often be distinguished by characteristic dark markings on the carapace, while the other two species are more difficult to separate, therefore examination of genitalia is required for their reliable identification ( Bee *et al.*, 2020). However, the season of maturity differs for the two species, which can be helpful in distinguishing between them (although not fully reliable): mature *M. segmentata* appears from August to October (mid-August to December according to ( Toft, 1983a), while *M. mengei* is mostly found from April to July ( Bee *et al.*, 2020; Roberts, 1995). All three species are common and widespread in Britain, and can be found in variety of habitats, on vegetation and structures from ground level to about 2 m high ( Roberts, 1995). *Metellina* spiders build orb webs with a radial frame supporting a spiral of sticky silk for catching the prey ( Álvarez-Padilla & Hormiga, 2011).

Adult size in *M. segmentata* usually ranges from 4–6 mm for males and 4–8 mm for females ( Bee *et al.*, 2020), with females being markedly heavier, and males having distinctively longer legs and a broader prosoma ( Prenter *et al.*, 1995). This species is also known to have variable body size in response to environmental conditions (for example, specimens in woodlands are smaller than those found in nearby clearings), while also displaying size decrease along a latitudinal gradient, being smaller in size towards the north of Britain ( Toft, 1983b).

*Metellina segmentata* has an annual life cycle. It overwinters as an egg, which then hatches around mid-June. The juveniles grow fast, going through four instars, and they mature in approximately two months ( Toft, 1983b). The juvenile spiders can usually be separated from the other two closely related species using abdominal pattern and markings on the cephalothorax ( Toft, 1983a).

The adults mature in late summer/autumn and display courtship behaviour, as well as mate-guarding behaviour, in which male can be often found guarding the orb web of a female (which was the case for the specimens used for this study). The females usually receive the interest of several males, while males typically can only guard a single female. Larger, fecund females appear to be more attractive to males and are guarded more viciously by males. Also, larger males have a higher success rate when competing with smaller opponents. The contest is usually resolved by assessment ( Hack *et al.*, 1997; Prenter *et al.*, 2003). The male waits for the female to capture and bite a prey before entering the web to copulate. This is likely to reduce the risk of becoming predated himself ( Prenter *et al.*, 1994).

*Metellina segmentata* was for a long time placed in the Metidae family, but it is now consolidated in the family Tetragnathiidae, which is monophyletic ( Álvarez-Padilla *et al.*, 2009). The phylogeny of this group was addressed using direct optimization methods, of multigene DNA sequences and the morphological and behavioural data by Álvarez-Padilla *et al*. (2009) and Álvarez-Padilla & Hormiga (2011). The monophyly of genus *Metellina* has not been tested as of yet ( Álvarez-Padilla & Hormiga, 2011).

The high-quality genome of *Metellina segmentata* was sequenced as part of the Darwin Tree of Life Project, a collaborative effort to sequence all named eukaryotic species in the Atlantic Archipelago of Britain and Ireland. Here we present a chromosomally complete genome sequence for *Metellina segmentata*, based on one male and one female specimen from Oxford.

## Genome sequence report

**Figure 1.**
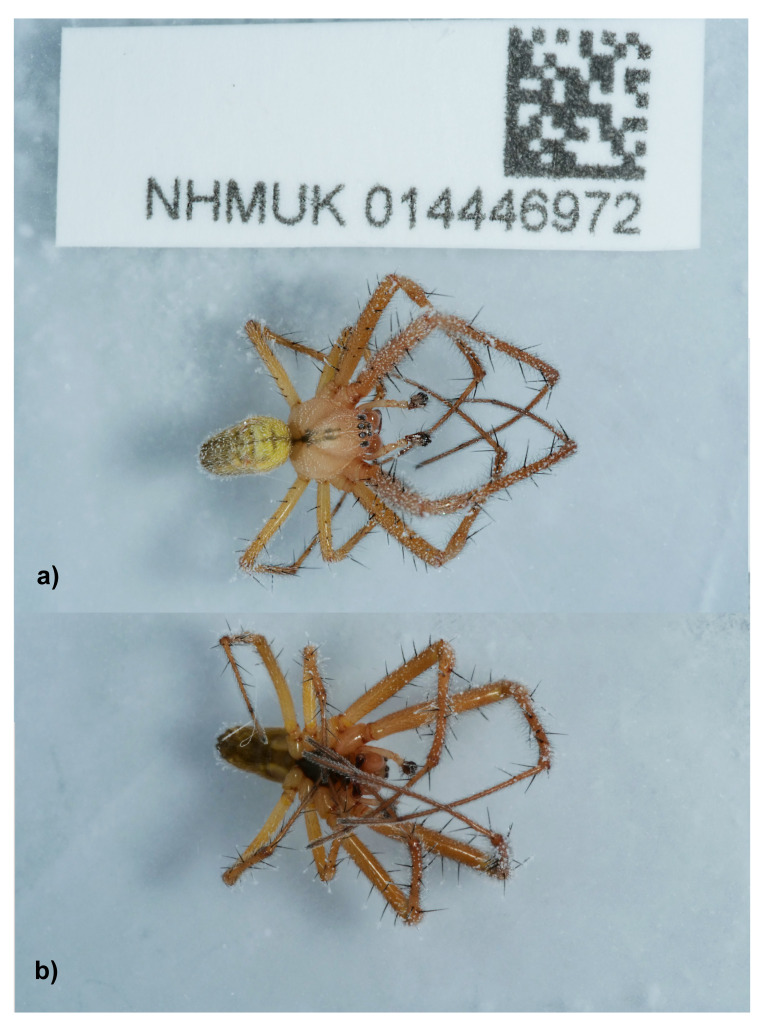
Photographs of *Metellina segmentata* (qqMetSegm1) taken during sample preservation and processing **a**) The specimen in dorsal view. **b**) The specimen in ventral view.

The genome was sequenced from one male *Metellina segmentata* ( Figure 1) collected from Louie Memorial Fields, Oxford, England (51.74, –1.30). A total of 27-fold coverage in Pacific Biosciences single-molecule HiFi long reads was generated. Primary assembly contigs were scaffolded with chromosome conformation Hi-C data. Manual assembly curation corrected 58 missing joins or mis-joins and removed seven haplotypic duplications, reducing the assembly length by 0.21% and the scaffold number by 11.88%, and increasing the scaffold N50 by 1.93%.

**Figure 2.**
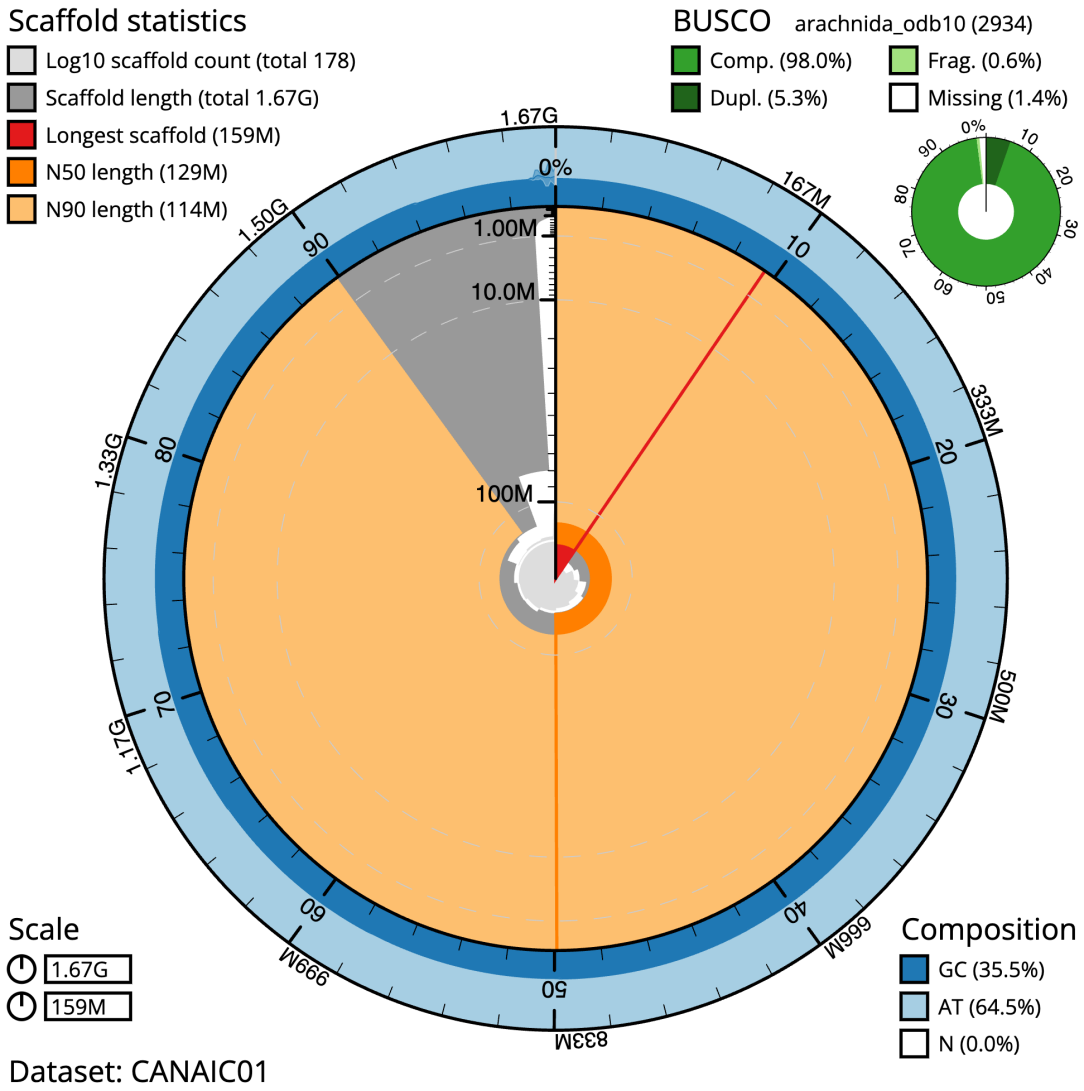
Genome assembly of *Metellina segmentata*, qqMetSegm1.1: metrics. The BlobToolKit Snailplot shows N50 metrics and BUSCO gene completeness. The main plot is divided into 1,000 size-ordered bins around the circumference with each bin representing 0.1% of the 1,665,125,926 bp assembly. The distribution of scaffold lengths is shown in dark grey with the plot radius scaled to the longest scaffold present in the assembly (158,549,065 bp, shown in red). Orange and pale-orange arcs show the N50 and N90 scaffold lengths (129,427,594 and 114,238,049 bp), respectively. The pale grey spiral shows the cumulative scaffold count on a log scale with white scale lines showing successive orders of magnitude. The blue and pale-blue area around the outside of the plot shows the distribution of GC, AT and N percentages in the same bins as the inner plot. A summary of complete, fragmented, duplicated and missing BUSCO genes in the arachnida_odb10 set is shown in the top right. An interactive version of this figure is available at https://blobtoolkit.genomehubs.org/view/qqMetSegm1.1/dataset/CANAIC01/snail..

The final assembly has a total length of 1,665.1 Mb in 178 sequence scaffolds with a scaffold N50 of 129.4 Mb ( Table 1). Most (98.99%) of the assembly sequence was assigned to 13 chromosomal-level scaffolds, representing 11 autosomes and the X1 and X2 sex chromosomes. Chromosome-scale scaffolds confirmed by the Hi-C data are named in order of size ( Figure 2– Figure 5; Table 2). While not fully phased, the assembly deposited is of one haplotype. Contigs corresponding to the second haplotype have also been deposited. The mitochondrial genome was also assembled and can be found as a contig within the multifasta file of the genome submission.

**Table 1.** Genome data for *Metellina segmentata*, qqMetSegm1.1.

Project accession data		
Assembly identifier	qqMetSegm1.1	
Species	*Metellina segmentata*	
Specimen	qqMetSegm1	
NCBI taxonomy ID	94026	
BioProject	PRJEB55727	
BioSample ID	SAMEA7696460	
Isolate information	qqMetSegm1, male, whole organism (DNA sequencing) qqMetSegm2, female, whole organism (Hi-C scaffolding)	
Assembly metrics *		*Benchmark*
Consensus quality (QV)	59.5	*≥ 50*
*k*-mer completeness	100%	*≥ 95%*
BUSCO **	C:98.0%[S:92.6%,D:5.3%], F:0.6%,M:1.4%,n:2,934	*C ≥ 95%*
Percentage of assembly mapped to chromosomes	98.99%	*≥ 95%*
Sex chromosomes	X _1_ and X _2_	*localised homologous pairs*
Organelles	Mitochondrial genome assembled	*complete single alleles*
Raw data accessions		
PacificBiosciences SEQUEL II	ERR10168718–ERR10168720	
Hi-C Illumina	ERR10149548	
Genome assembly		
Assembly accession	GCA_947359465.1	
*Accession of alternate haplotype*	GCA_947359365.1	
Span (Mb)	1,665.1	
Number of contigs	1,060	
Contig N50 length (Mb)	3.4	
Number of scaffolds	178	
Scaffold N50 length (Mb)	129.4	
Longest scaffold (Mb)	158.6	

* Assembly metric benchmarks are adapted from column VGP-2020 of “Table 1: Proposed standards and metrics for defining genome assembly quality” from ( Rhie *et al.*, 2021). ** BUSCO scores based on the arachnida_odb10 BUSCO set using v5.3.2. C = complete [S = single copy, D = duplicated], F = fragmented, M = missing, n = number of orthologues in comparison. A full set of BUSCO scores is available at https://blobtoolkit.genomehubs.org/view/qqMetSegm1.1/dataset/CANAIC01/busco.

**Figure 3.**
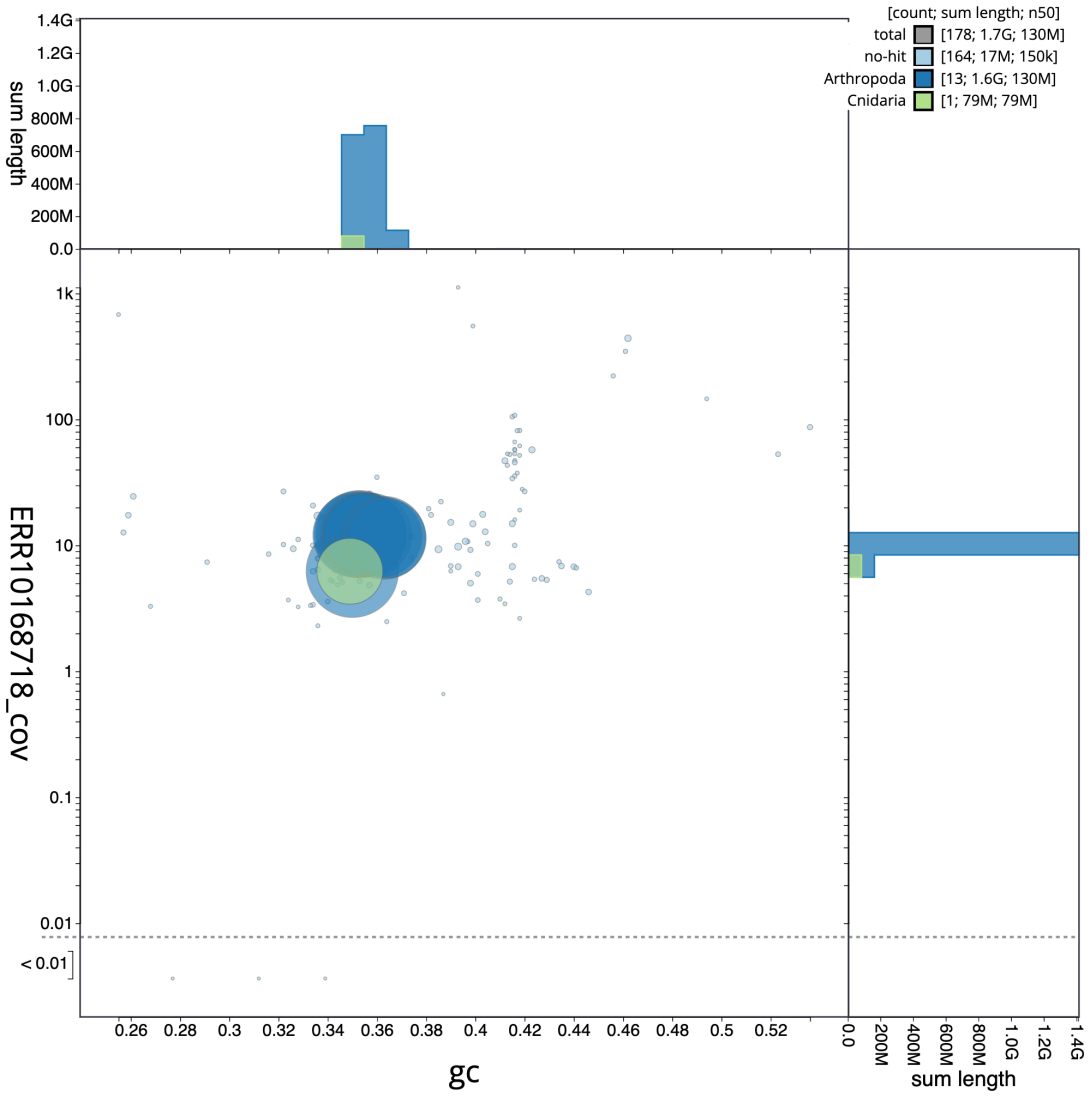
Genome assembly of *Metellina segmentata*, qqMetSegm1.1: BlobToolKit GC-coverage plot. Scaffolds are coloured by phylum. Circles are sized in proportion to scaffold length. Histograms show the distribution of scaffold length sum along each axis. An interactive version of this figure is available at https://blobtoolkit.genomehubs.org/view/qqMetSegm1.1/dataset/CANAIC01/blob.

**Figure 4.**
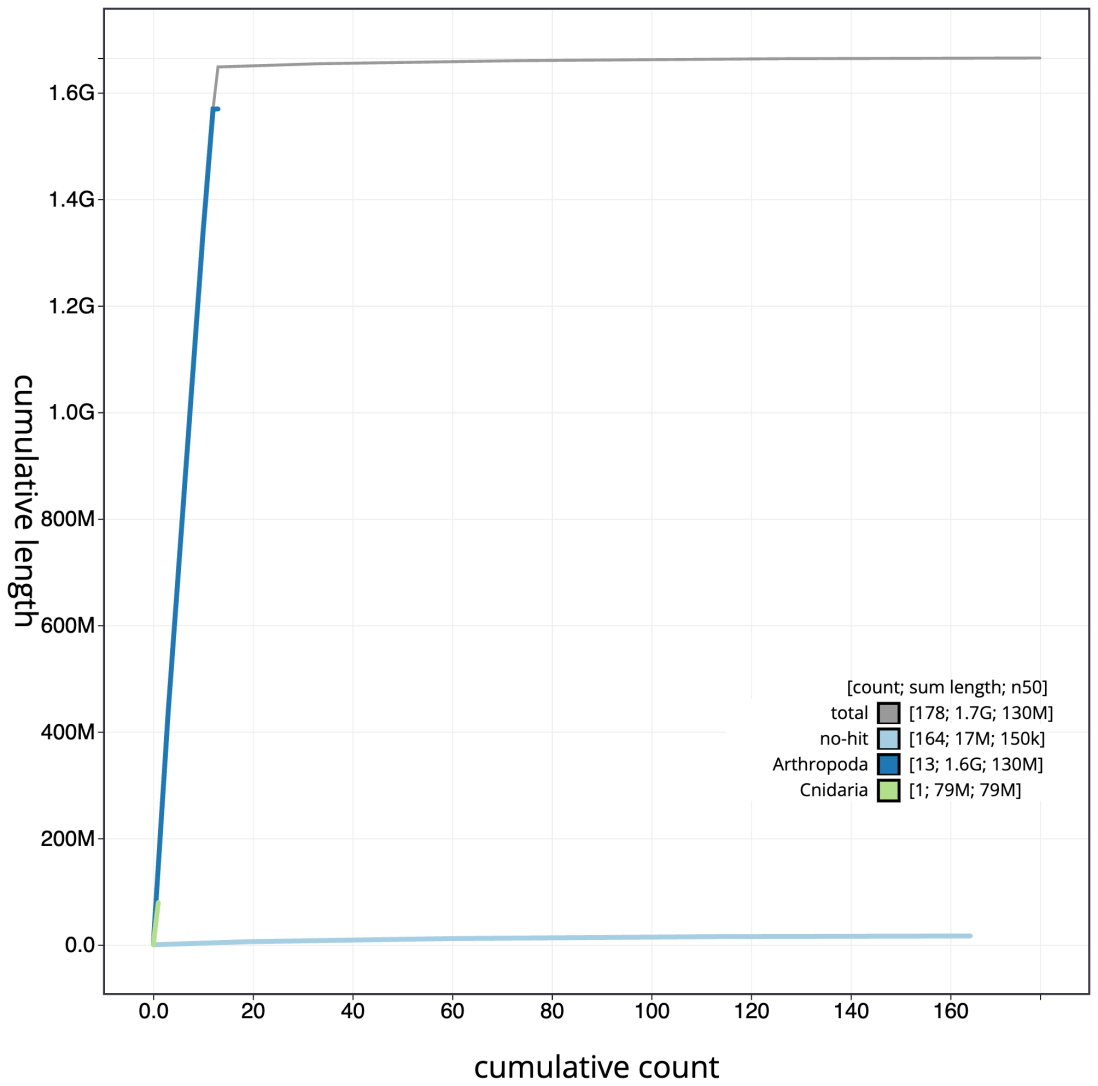
Genome assembly of *Metellina segmentata*, qqMetSegm1.1: BlobToolKit cumulative sequence plot. The grey line shows cumulative length for all scaffolds. Coloured lines show cumulative lengths of scaffolds assigned to each phylum using the buscogenes taxrule. An interactive version of this figure is available at https://blobtoolkit.genomehubs.org/view/qqMetSegm1.1/dataset/CANAIC01/cumulative.

**Figure 5.**
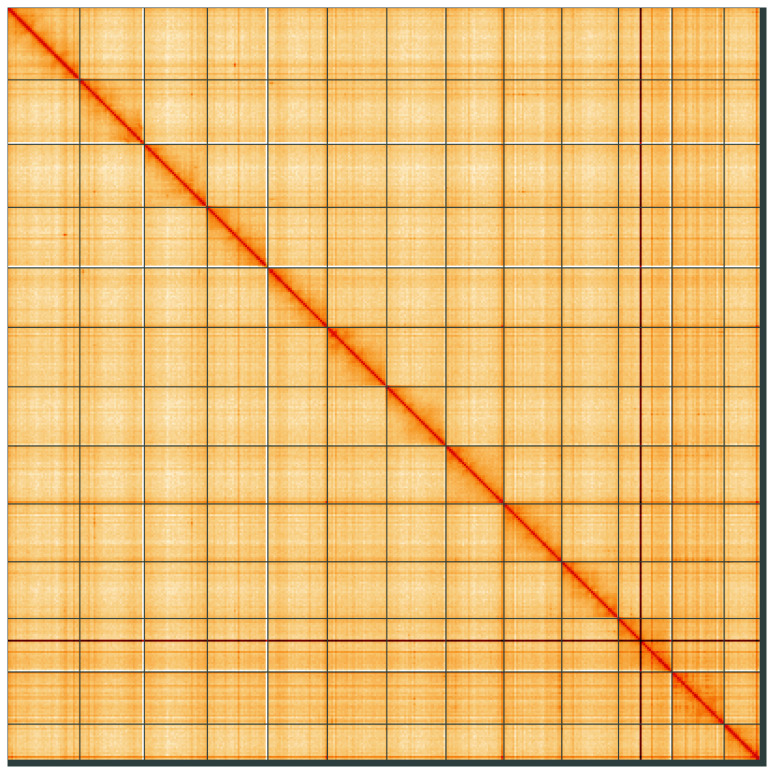
Genome assembly of *Metellina segmentata*, qqMetSegm1.1: Hi-C contact map of the qqMetSegm1.1 assembly, visualised using HiGlass. Chromosomes are shown in order of size from left to right and top to bottom. An interactive version of this figure may be viewed at https://genome-note-higlass.tol.sanger.ac.uk/l/?d=M2Yi5bBqSUWTs_1YLisBxg.

**Table 2.** Chromosomal pseudomolecules in the genome assembly of *Metellina segmentata*, qqMetSegm1.

INSDC accession	Chromosome	Size (Mb)	GC%
OX375873.1	1	141.1	35.3
OX375872.1	X1	158.55	35
OX375874.1	2	137.99	35.2
OX375875.1	3	132.53	35.4
OX375884.1	X2	79.13	34.9
OX375876.1	4	130.32	35.5
OX375877.1	5	130.21	35.7
OX375878.1	6	129.43	35.1
OX375879.1	7	126.98	35.5
OX375880.1	8	126.56	36.3
OX375881.1	9	124.24	35.5
OX375882.1	10	117.07	36.2
OX375883.1	11	114.24	36.4
OX375885.1	MT	0.02	25.5

The estimated Quality Value (QV) of the final assembly is 59.5 with *k*-mer completeness of 100%, and the assembly has a BUSCO v5.3.2 completeness of 98.0% (single = 92.6%, duplicated = 5.3%), using the arachnida_odb10 reference set ( *n* = 2,934).

Metadata for specimens, spectral estimates, sequencing runs, contaminants and pre-curation assembly statistics can be found at https://links.tol.sanger.ac.uk/species/94026.

## Methods

### Sample acquisition and nucleic acid extraction

One female (NHMUK014446973, qqMetSegm2) and one male (NHMUK014446972, qqMetSegm1) of *Metellina segmentata* ( Figure 1) were hand-picked from Louie Memorial Fields, Oxford, England (51.74, –1.30) on 13 September 2020 and identified by Sergio Henriques. The specimens were snap-frozen using dry ice. The tissue samples taken from them were stored in a CoolRack prior to genome sequencing.

DNA was extracted at the Tree of Life laboratory, Wellcome Sanger Institute (WSI). The qqMetSegm1 sample was weighed and dissected on dry ice. Whole organism tissue was disrupted using a Nippi Powermasher fitted with a BioMasher pestle. High molecular weight (HMW) DNA was extracted using the Qiagen MagAttract HMW DNA extraction kit. HMW DNA was sheared into an average fragment size of 12–20 kb in a Megaruptor 3 system with speed setting 30. Sheared DNA was purified by solid-phase reversible immobilisation using AMPure PB beads with a 1.8X ratio of beads to sample to remove the shorter fragments and concentrate the DNA sample. The concentration of the sheared and purified DNA was assessed using a Nanodrop spectrophotometer and Qubit Fluorometer and Qubit dsDNA High Sensitivity Assay kit. Fragment size distribution was evaluated by running the sample on the FemtoPulse system.

### Sequencing

Pacific Biosciences HiFi circular consensus DNA sequencing libraries were constructed according to the manufacturers’ instructions. DNA sequencing was performed by the Scientific Operations core at the WSI on Pacific Biosciences SEQUEL II (HiFi) instrument. Hi-C data were also generated from whole organism tissue of qqMetSegm2 using the Arima2 kit and sequenced on the Illumina NovaSeq 6000 instrument.

### Genome assembly, curation and evaluation

Assembly was carried out with Hifiasm ( Cheng *et al.*, 2021) and haplotypic duplication was identified and removed with purge_dups ( Guan *et al.*, 2020). The assembly was then scaffolded with Hi-C data ( Rao *et al.*, 2014) using YaHS ( Zhou *et al.*, 2023). The assembly was checked for contamination as described previously ( Howe *et al.*, 2021). Manual curation was performed using HiGlass ( Kerpedjiev *et al.*, 2018) and Pretext ( Harry, 2022). The mitochondrial genome was assembled using MitoHiFi ( Uliano-Silva *et al.*, 2022), which runs MitoFinder ( Allio *et al.*, 2020) or MITOS ( Bernt *et al.*, 2013) and uses these annotations to select the final mitochondrial contig and to ensure the general quality of the sequence.

A Hi-C map for the final assembly was produced using bwa-mem2 ( Vasimuddin *et al.*, 2019) in the Cooler file format ( Abdennur & Mirny, 2020). To assess the assembly metrics, the *k*-mer completeness and QV consensus quality values were calculated in Merqury ( Rhie *et al.*, 2020). This work was done using Nextflow ( Di Tommaso *et al.*, 2017) DSL2 pipelines “sanger-tol/readmapping” ( Surana *et al.*, 2023a) and “sanger-tol/genomenote” ( Surana *et al.*, 2023b). The genome was analysed within the BlobToolKit environment ( Challis *et al.*, 2020) and BUSCO scores ( Manni *et al.*, 2021; Simão *et al.*, 2015) were calculated.

Table 3 contains a list of relevant software tool versions and sources.

**Table 3.** Software tools: versions and sources.

Software tool	Version	Source
BlobToolKit	4.0.7	https://github.com/ blobtoolkit/blobtoolkit
BUSCO	5.3.2	https://gitlab.com/ezlab/ busco
Hifiasm	0.16.1-r375	https://github.com/ chhylp123/hifiasm
HiGlass	1.11.6	https://github.com/ higlass/higlass
Merqury	MerquryFK	https://github. com/thegenemyers/ MERQURY.FK
MitoHiFi	2	https://github.com/ marcelauliano/MitoHiFi
PretextView	0.2	https://github.com/wtsi- hpag/PretextView
purge_dups	1.2.3	https://github.com/ dfguan/purge_dups
sanger-tol/ genomenote	v1.0	https://github.com/ sanger-tol/genomenote
sanger-tol/ readmapping	1.1.0	https://github.com/ sanger-tol/readmapping/ tree/1.1.0
YaHS	yahs-1.1.91eebc2	https://github.com/c- zhou/yahs

### Ethics and compliance issues

The materials that have contributed to this genome note have been supplied by a Darwin Tree of Life Partner. The submission of materials by a Darwin Tree of Life Partner is subject to the Darwin Tree of Life Project Sampling Code of Practice. By agreeing with and signing up to the Sampling Code of Practice, the Darwin Tree of Life Partner agrees they will meet the legal and ethical requirements and standards set out within this document in respect of all samples acquired for, and supplied to, the Darwin Tree of Life Project. Each transfer of samples is further undertaken according to a Research Collaboration Agreement or Material Transfer Agreement entered into by the Darwin Tree of Life Partner, Genome Research Limited (operating as the Wellcome Sanger Institute), and in some circumstances other Darwin Tree of Life collaborators.

## Data Availability

European Nucleotide Archive: *Metellina segmentata* (autumn spider). Accession number PRJEB55727; https://identifiers.org/ena.embl/PRJEB55727 ( Wellcome Sanger Institute, 2022). The genome sequence is released openly for reuse. The *Metellina segmentata* genome sequencing initiative is part of the Darwin Tree of Life (DToL) project. All raw sequence data and the assembly have been deposited in INSDC databases. The genome will be annotated using available RNA-Seq data and presented through the Ensembl pipeline at the European Bioinformatics Institute. Raw data and assembly accession identifiers are reported in Table 1.

## Author information

Members of the Natural History Museum Genome Acquisition Lab are listed here:

https://doi.org/10.5281/zenodo.4790042.

Members of the Darwin Tree of Life Barcoding collective are listed here: https://doi.org/10.5281/zenodo.4893703.

Members of the Wellcome Sanger Institute Tree of Life programme are listed here: https://doi.org/10.5281/zenodo.4783585.

Members of Wellcome Sanger Institute Scientific Operations: DNA Pipelines collective are listed here: https://doi.org/10.5281/zenodo.4790455.

Members of the Tree of Life Core Informatics collective are listed here: https://doi.org/10.5281/zenodo.5013541.

Members of the Darwin Tree of Life Consortium are listed here: https://doi.org/10.5281/zenodo.4783558.
