# Supplementary material for: The genome sequence of the Autumn Spider, Metellina segmentata (Clerck, 1757)
Source: Wellcome Open Res. 2023 May 16;8:221. [Version 1] doi: 10.12688/wellcomeopenres.19435.1 (PMC11519618; doi:10.12688/wellcomeopenres.19435.1)
Supplement: Peer review history

# Reviewer report: Reviewer response for version 1

**DOI:** 10.21956/wellcomeopenres.21531.r104313

**Published:** 2024-10-28

**Authors:** Marc Domènech

**Affiliations:** 1 Universitat de Barcelona, Barcelona, Catalonia, Spain

This report presents the chromosome-scale genome of *Metellina segmentata*. The accession numbers and additional information are provided. The results are well structured. It is not completely clear in the manuscript how the specimens were identified. Was it based on their genitalia and/or later confirmed genetically?

 This new high-quality genomic data will be useful for future studies assessing the phylogeny of spiders or arachnids in general.

Are sufficient details of methods and materials provided to allow replication by others?

Yes

Is the rationale for creating the dataset(s) clearly described?

Yes

Are the datasets clearly presented in a useable and accessible format?

Yes

Are the protocols appropriate and is the work technically sound?

Yes

Reviewer Expertise:

Arachnid taxonomy, phylogenetics

I confirm that I have read this submission and believe that I have an appropriate level of expertise to confirm that it is of an acceptable scientific standard.

# Reviewer report: Reviewer response for version 1

**DOI:** 10.21956/wellcomeopenres.21531.r105575

**Published:** 2024-10-28

**Authors:** Frederik Hendrickx

**Affiliations:** 1 Ghent University, Royal Belgian Institute of Natural Sciences, Brussels, Belgium

This paper presents the genome assembly of the spider *Metellina segmentata*. With few high-quality, chromosome-level spider genomes currently available, this work represents a valuable addition to the field, enhancing our understanding of spidroin gene evolution, spider and arthropod phylogenetics, venom protein evolution, and developmental biology in arthropods, among other topics.

 The genome assembly is of high quality, thoroughly curated, and 99% of the assembled sequences have been assigned to the 13 chromosomes. While the raw data and assembled sequences are accessible to other researchers via ENA, reproducibility could be further strengthened by providing bioinformatic scripts used in the analysis pipeline, as the current availability includes only the software employed.

Are sufficient details of methods and materials provided to allow replication by others?

Partly

Is the rationale for creating the dataset(s) clearly described?

Yes

Are the datasets clearly presented in a useable and accessible format?

Yes

Are the protocols appropriate and is the work technically sound?

Yes

Reviewer Expertise:

Spider genome evolution

I confirm that I have read this submission and believe that I have an appropriate level of expertise to confirm that it is of an acceptable scientific standard.

# Reviewer report: Reviewer response for version 1

**DOI:** 10.21956/wellcomeopenres.21531.r57569

**Published:** 2023-05-18

**Authors:** Nadia Ayoub

**Affiliations:** 1 Department of Biology, Washington and Lee University, Lexington, Virginia, USA

This data note reports the chromosome-level assembly of the genome of a tetragnathid spider. While other genera in the Tetragnathidae have genome sequences, *Metellina* does not yet have a published genome. The genome reported here will be of excellent use to the genomics community, especially those interested in spider evolution. The report provides sufficient detail of methods for sequencing and assembly. The assembly is already publicly available for download through NCBI and the appropriate accession numbers are clearly provided in the report. The genome would be even more useful with an annotation, but I appreciate that the assembly is available to search and annotate.

Are sufficient details of methods and materials provided to allow replication by others?

Yes

Is the rationale for creating the dataset(s) clearly described?

Yes

Are the datasets clearly presented in a useable and accessible format?

Yes

Are the protocols appropriate and is the work technically sound?

Yes

Reviewer Expertise:

Spider silk; molecular evolution; transcriptomics

I confirm that I have read this submission and believe that I have an appropriate level of expertise to confirm that it is of an acceptable scientific standard.
